# Reciprocal cross-feeding between bacteria can limit the emergence of metabolic dependencies

**DOI:** 10.1128/aem.00363-26

**Published:** 2026-05-12

**Authors:** Ying-Chih Chuang, Megan G. Behringer, Gillian E. Patton, Jordan T. Bird, Jeffrey L. Mazny, Jennifer R. Gliessman, Crystal E. Love, Ankur B. Dalia, James B. McKinlay

**Affiliations:** 1 Department of Biology, Indiana University 1772, Bloomington, Indiana, USA; 2 Biochemistry Program, Indiana University 1772, Bloomington, Indiana, USA; 3 Department of Biological Sciences, Vanderbilt University 5718, Nashville, Tennessee, USA; 4 Battelle Memorial Institute 42786, Columbus, Ohio, USA; Danmarks Tekniske Universitet, Kgs. Lyngby, Denmark

**Keywords:** adaptive gene loss, microbial physiology, microbial ecology, hydrogen, siderophores, purines, *Rhodopseudomonas*, phototrophy, fermentation, metabolism

## Abstract

Cross-feeding is prevalent in microbial communities. Through time, cross-feeding is thought to enrich for loss-of-function mutations, thereby creating or reinforcing dependencies between community members. However, few studies have compared how cross-feeding affects the evolutionary trajectory of partners compared to monoculture conditions. Here, we compared mutations that were differentially enriched in bacterial monocultures versus cocultures pairing phototrophic *Rhodopseudomonas palustris* with fermentative *Escherichia coli* in an obligate cross-feeding relationship based on the exchange of nitrogen and carbon for 650–800 generations. Opposite trends for the number of differentially enriched mutations were observed for each species; *R. palustris* accumulated more unique mutations in monoculture, whereas *E. coli* accumulated more unique mutations in co-culture. Contrary to expectations, the emergence of additional dependencies was observed in monoculture, but not in coculture, even though additional layers of cross-feeding involving iron and adenine were present in coculture. We reasoned that iron and adenine cross-feeding occurred at levels sufficient to repress gene expression in the recipient, thereby promoting gene retention by lowering gene cost. We also observed that *E. coli* acquired mutations in coculture that were suggestive of enhanced adenine uptake, whereas *R. palustris* gained the ability to oxidize H_2_, but only in monoculture. Thus, the influence of cross-feeding on evolutionary trajectories can vary with organisms and conditions, and there are situations where cross-feeding can limit, rather than promote, emergent dependencies.

**IMPORTANCE:**

Bacteria commonly engage in cross-feeding, where nutrients are transferred between neighbors. Cross-feeding is thought to alleviate energy expenditures for genes whose role can be met by cross-fed nutrients, leading to eventual gene loss. However, few examples have been documented, especially in comparison to monocultures that lack a cross-feeding partner. We grew cocultures pairing phototrophic *Rhodopseudomonas palustris* with fermentative *Escherichia coli* alongside corresponding monocultures for 650–800 generations. While coculture conditions required obligate exchange of nitrogen and carbon, additional cross-feeding of adenine and iron likely occurred. Contrary to expectations, dependencies for iron and unknown compounds emerged in monocultures, but expected iron and adenine dependencies were not observed in cocultures. Low expression of iron scavenging and adenine synthesis genes in cocultures suggested that cross-feeding repressed expression, thereby lowering gene cost. Thus, although cross-feeding can sometimes make costly genes dispensable, there are also cases where cross-feeding lowers gene cost, thereby promoting gene retention.

## INTRODUCTION

Microbial metabolism shapes local chemical environments in ways that elicit regulated responses from neighboring cells or even select for adaptive mutations. In some cases, extracellular metabolites from producer cells can be used as nutrients by neighboring recipient cells, establishing cross-feeding (1). Cross-feeding can lead to obligate dependencies through adaptive loss-of-function (LOF) mutations, when the benefit of exploiting an extracellular metabolite outweighs the cost of retaining a biosynthetic gene, as described in the Black Queen Hypothesis (BQH) (2). The BQH is commonly used to explain the prevalence of auxotrophs, which cannot synthesize one or more essential nutrients (3, 4). Indeed, several groups observed that auxotrophs with biosynthetic gene deletions have a fitness advantage over their prototrophic counterparts when corresponding biosynthetic precursors were provided (5–9).

While cross-feeding can promote adaptive LOF mutations, the enrichment of LOF mutants is also potentially constrained during cross-feeding by several factors. For example, the energetic cost of N_2_ fixation, converting N_2_ into 2 NH_4_^+^ via nitrogenase, was predicted to be under pressure for gene loss because NH_4_^+^ can escape producer cells and potentially enable a higher growth rate for recipients (2). However, we found that when recipients depended on NH_4_^+^ from a producer, the recipient growth rate was lower than that with N_2_ because the extracellular NH_4_^+^ concentration was low, even when producers were engineered to excrete NH_4_^+^ (10). Based on these growth kinetics, a nitrogenase LOF mutant would not have a growth advantage and thus would not be enriched. Moreover, as was documented in studies of exploitive “cheater” mutants that lack iron-scavenging siderophores, iron availability repressed siderophore gene expression and thereby limited the benefit of LOF mutations by lowering gene cost (11–13); most of a gene’s cost comes from its expression (14–19). Thus, repressing gene expression could have similar cost-savings as LOF mutations and thereby decrease selective pressure for LOF mutants.

Previously, we engineered cocultures of phototrophic *R. palustris* and fermentative *E. coli* to study the mechanisms and evolution of obligate cross-feeding (20–24). Using anoxic conditions, *E. coli* ferments glucose into organic acids that provide essential carbon to *R. palustris* while *R. palustris* converts N_2_ into NH_4_^+^, providing essential nitrogen to *E. coli*; NH_4_^+^ excretion is due to an engineered NifA* mutation (20). The two species are not believed to interact in nature, providing an opportunity to observe how the relationship evolves without pre-adaptation (21). Although the coculture was engineered, and verified, to rely on NH_4_^+^ cross-feeding (20–24), other interaction layers exist. For example, *R. palustris* externalizes enough of the purine, adenine, to support an *E. coli* adenine auxotroph population (25, 26). Transcriptomic analyses also showed that *R. palustris* downregulates siderophores gene expression in coculture, suggesting that it can acquire iron from *E. coli* (22). How such secondary interactions affect the evolutionary trajectory of each species is unknown.

Here, we compared differential mutation accumulation in *E. coli* and *R. palustris* monocultures versus cocultures for >600 generations. The number of differentially enriched mutations in monocultures versus cocultures was opposite for each organism. For several mutations, we performed a preliminary investigation to gain insights into why they were differentially enriched. We also explored why some anticipated dependencies were not observed. In some cases, we correlated a lack of dependency emergence in coculture to inhibitory effects of cross-feeding on gene expression. Our results offer diverse insights into how cross-feeding influences evolutionary trajectories and caution against general expectations for dependencies through LOF mutations.

## RESULTS AND DISCUSSION

### *R. palustris* and *E. coli* can coexist for hundreds of generations

**Fig 1:**
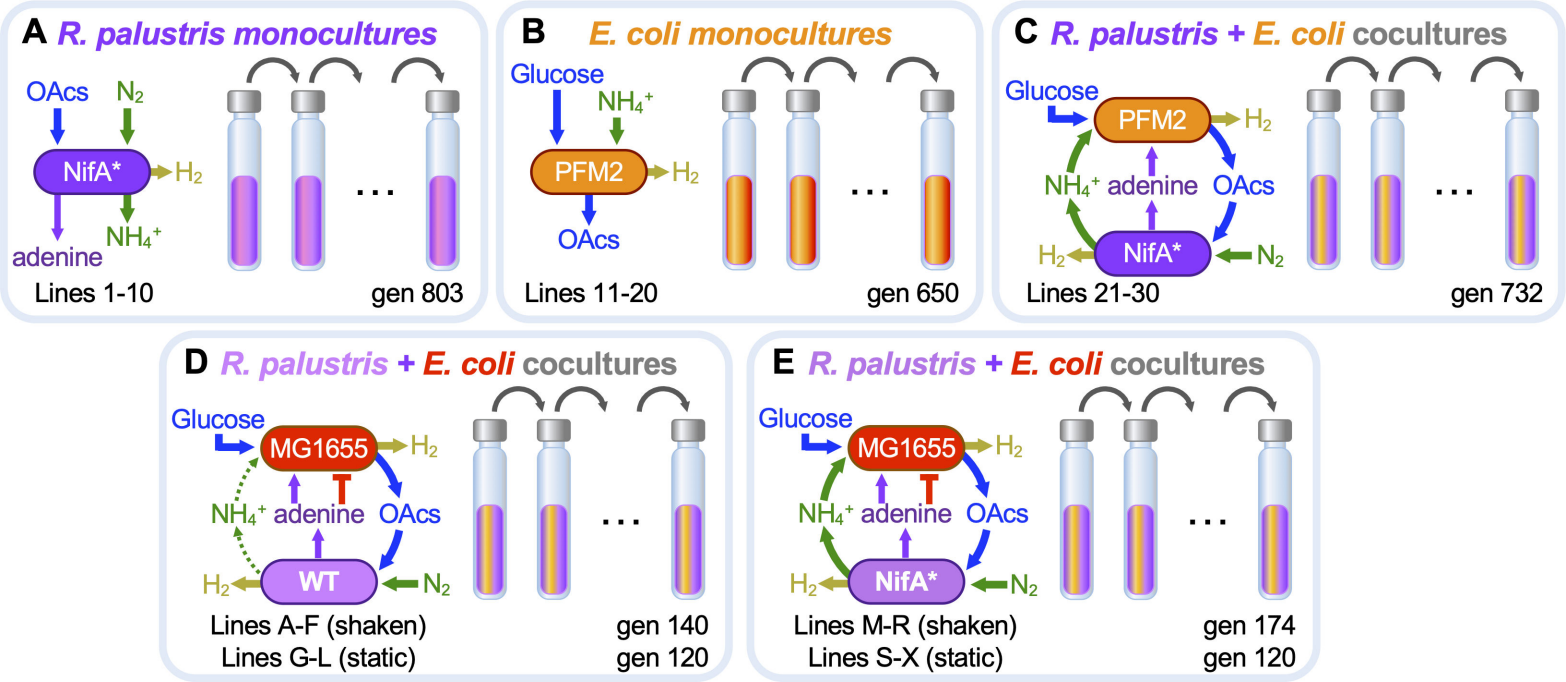
Long-term anaerobic monoculture and coculture conditions. (**A–C**) All cultures were laid flat without shaking and were constantly illuminated. (**A**) Phototrophic *R. palustris* NifA* (CGA676) was fed a mixture of organic acids (OAcs), ethanol, and NaHCO_3_ to mimic carbon sources available from *E. coli* fermentation products; glucose was also provided but was not consumed by *R. palustris*. The NifA* mutation causes NH_4_^+^ excretion during N_2_ fixation. H_2_ is also produced as a co-product of N_2_ fixation. *R. palustris* also excretes adenine independent of the NifA* mutation. (**B**) *E. coli* PFM2 was fed 10 mM NH_4_Cl and 10 mM glucose, which it fermented to a mixture of OAcs, ethanol, CO_2_, and H_2_. (**C**) Cocultures were fed 25 mM glucose. Growth of each species is dependent on the exchange of NH_4_^+^ and OAcs. (**D and E**) Previously studied long-term cocultures of *E. coli* MG1655 and hydrogenase-deficient (*ΔhupS*) strains of *R. palustris* with either wild-type NifA (**D**; WT; CGA4001) or a NifA* mutation (**E**; CGA4003). These cocultures were fed 50 mM glucose. Further details can be found elsewhere (21). PFM2 is an *E. coli* MG1655 derivative with repaired *rpoS* and *rph* genes.

To compare the evolutionary trajectory of *R. palustris* and *E. coli* in monoculture versus coculture, we established 10 replicate long-term, batch, anaerobic cultures in each of three conditions: (i) monocultures of phototrophic, N_2_-fixing, NH_4_^+^-excreting *R. palustris* NifA* (CGA676) (27), supplied with a mixture of organic acid salts (neutral pH), ethanol, and bicarbonate to mimic *E. coli* fermentation products (Fig. 1A); glucose was also provided, but *R. palustris* cannot consume glucose. (ii) Monocultures of fermentative *E. coli* PFM2, a MG1655 derivative with repaired *rpoS* and *rph* genes (28, 29), supplied with 10 mM glucose and 10 mM NH_4_Cl (Fig. 1B). (iii) Cocultures supplied with 25 mM glucose (Fig. 1C). *E. coli* monocultures had less glucose (10 mM) to avoid lethal accumulation of acidifying organic acids, strong selection for acid-resistant mutants, and to help ensure that the primary difference between monocultures and cocultures was the presence or absence of the partner species. Lethal acidification was not an issue in cocultures, despite using 25 mM glucose, due to organic acid consumption by *R. palustris* (20). All cultures were constantly illuminated, and 2.5% vol/vol was transferred every 7 days. All conditions included an exponential growth phase, but only monocultures experienced a nutrient-limited stationary phase. In cocultures, glucose and consumable organic acids remained at the time of transfer and likely supported growth; however, the growth was slow due to a low pH. Ultimately, all conditions were serially transferred for 650–803 generations (~5 generations per transfer; Fig. 1A through C). Herein, we also make occasional reference to previous long-term cocultures combining *E. coli* MG1655 and *R. palustris* strains with and without NifA* mutations (21).

**Fig 2:**
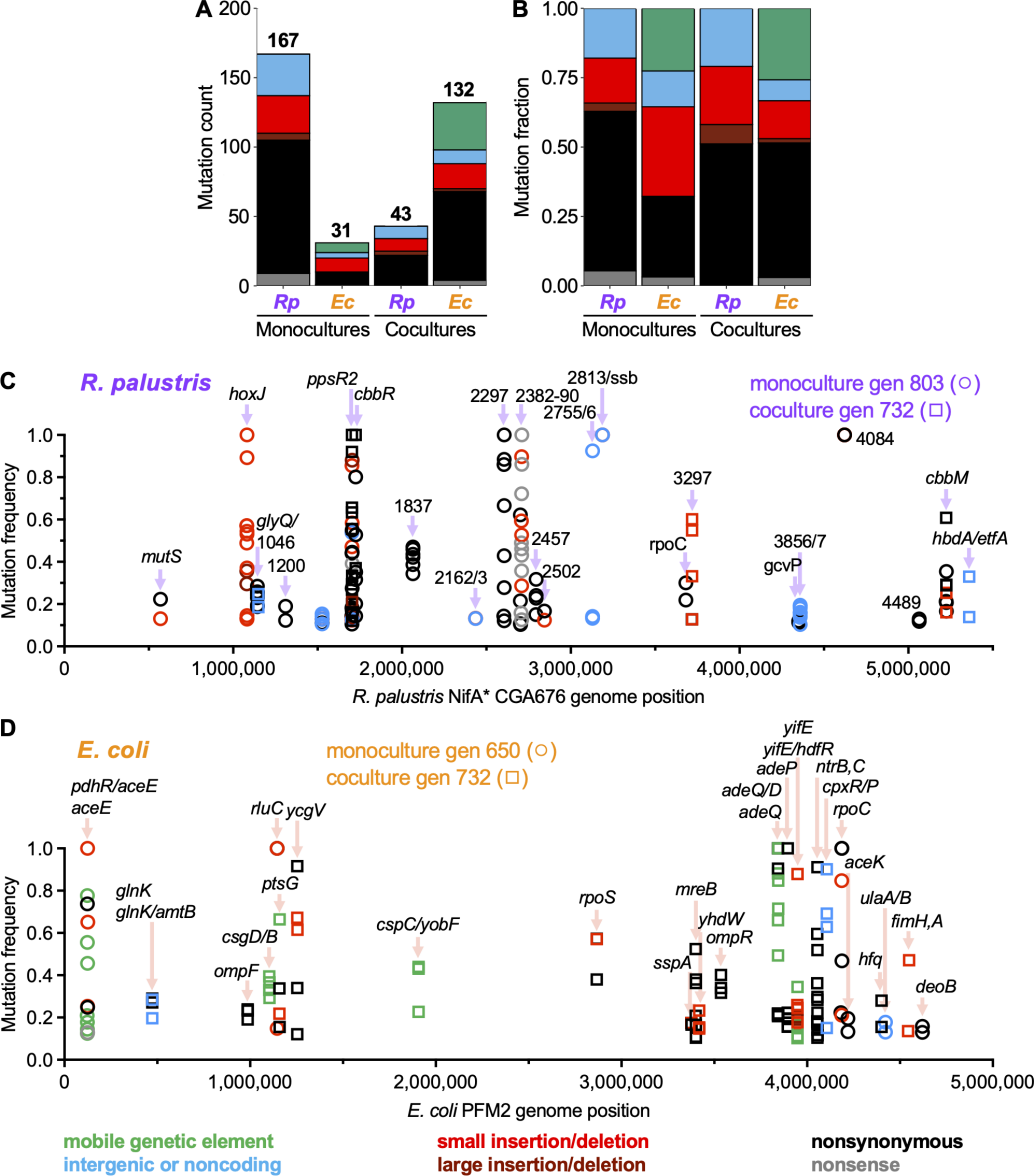
Differentially enriched mutations in long-term monocultures and cocultures. Mutation counts (**A**) and fractions (**B**) are the sum of mutations across all 10 replicates in each condition. To be counted, mutations had to meet the following criteria: (i) exclude synonymous mutations, (ii) exclude mutations that were common between monocultures and cocultures (different mutations in a common gene were allowed), (iii) must be observed in the last two sequenced time points (*R. palustris* (*Rp*) monoculture, generations 781 and 803; *E. coli* (*Ec*) monoculture, generations 500 and 650; coculture, generations 655 and 732), and (iv) must have a frequency > 0.1 in the last time point. (**C and D**) Each point represents a mutation in a replicate monoculture or coculture for *R. palustris* (**C**) or *E. coli* PFM2 (**D**). Many mutations in a common region generally mean that the region was mutated in replicate cultures. Occurrences of > 2 nearby mutations within a single replicate were rare (i.e., *adeQ/D* and *adeP* in *E. coli* replicate 30 are exceptions, Tables S1 to S4). Mutations are shown if they met the above criteria and if (v) the same gene or intergenic region was mutated in at least two replicates for a given condition. Generation: gen. Nonsynonymous mutations are SNPs that change the codon, resulting in a different amino acid. Nonsense mutations are SNPs that create a premature stop codon.

We sequenced gDNA from populations at several time points. We then compared mutations that were differentially enriched in monoculture versus coculture by applying the following criteria: (i) exclude synonymous mutations (i.e., single-nucleotide polymorphisms (SNPs) within codons that do not change amino acid composition), (ii) exclude mutations that were common between monoculture and cocultures (different mutations in a common gene were allowed), (iii) must be observed in the last two sequenced time points, and (iv) must have a frequency >0.1 in the last time point. These criteria resulted in a list of 167 mutations from *R. palustris* monocultures, 31 from *E. coli* monocultures, 43 from *R. palustris* in cocultures, and 132 from *E. coli* in cocultures (Fig. 2A; Tables S1 through S5). These counts include redundancy, where some mutations were observed in multiple replicate monocultures or cocultures. In all conditions, most mutations that met our criteria were nonsynonymous (i.e., SNPs that change a codon to code for a different amino acid), except for *E. coli* monocultures, where most mutations were small insertions/deletions (Fig. 2B). Mutations due to mobile genetic elements were only observed in *E. coli* (Fig. 2B). To narrow our focus, we added a fifth criterion of parallelism where the same gene or intergenic region must be mutated in at least two replicate cultures in the last two time points (Fig. 2C and D).

### Mutation accumulation trends in monoculture versus coculture differed for each species

The number of differentially enriched mutations in monoculture versus coculture was oppositely skewed for each species; *R. palustris* acquired more mutations in monoculture, whereas *E. coli* acquired more mutations in coculture (Fig. 2A and B). When considering commonly mutated genes and intergenic regions, 19 were mutated in monoculture vs. five in coculture for *R. palustris* (Fig. 2C), and six were mutated in monoculture vs. 19 in coculture for *E. coli* (Fig. 2D). Similar observations of cocultures leading to more mutations were made by another group who paired *E. coli* auxotrophs in reciprocal cross-feeding cocultures versus monocultures (30), but our *R. palustris* results suggest that such trends might not be generalizable.

We considered whether the higher mutation frequency in *R. palustris* monocultures was due to mutations in *mutS,* encoding a mismatch repair protein; replicates 2 and 7 had a *mutS* mutation that met our criteria, and most other replicate monocultures had a low-frequency *mutS* mutation at some point during the experiment, whereas *R. palustris mutS* mutations were not observed in coculture. However, the number of differentially enriched mutations was similar across replicate *R. palustris* monocultures (range: 8–23; Table S1), with only 6 of 21 mutations in replicate 2, and 11 of 23 mutations in replicate 7 potentially attributed to *mutS*, since they occurred at a lower frequency than the *mutS* mutation (*mutS* mutation frequency was 13% and 22% in replicates 2 and 7, respectively; Table S1). Thus, *mutS* mutations did not likely drive many of the other mutations.

Below, we explore why some dependencies that we expected to emerge in cocultures via LOF mutations were not observed. We then focus on genes and regions that were differentially mutated between monocultures and cocultures that were more likely to be gain-of-function mutations. Brief insights into other differentially enriched mutations are in the Supplemental Text, along with some *R. palustris* genes that were commonly mutated between monocultures and cocultures, suggesting a general benefit.

### Cross-feeding of adenine resulted in lower purine biosynthesis gene expression, but not in auxotrophic mutants

**Fig 3:**
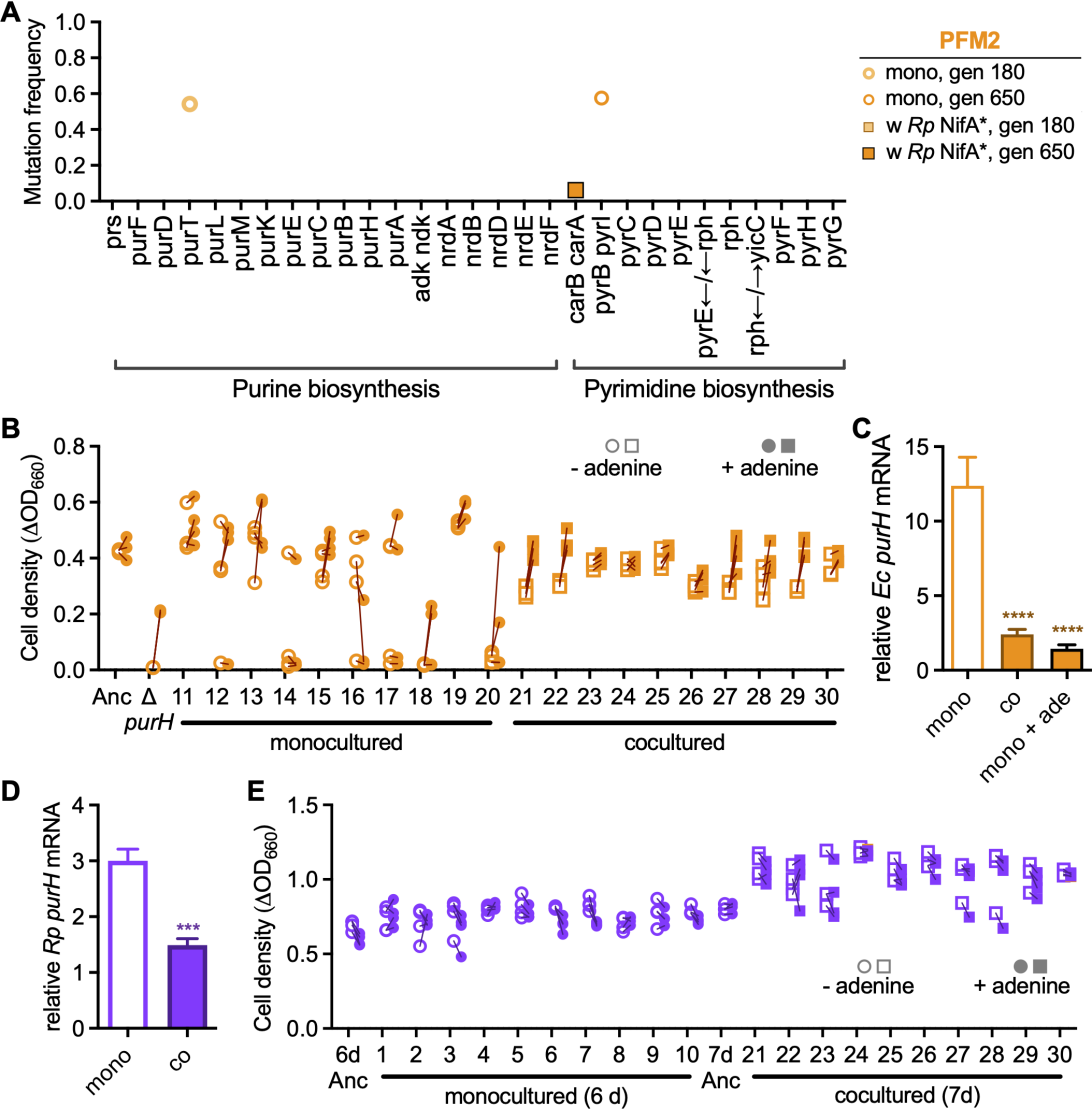
*E. coli* PFM2 adenine auxotrophs were not observed despite adenine availability in cocultures. (**A**) Each point represents an *E. coli* PFM2 mutation frequency in one replicate monoculture or coculture (Fig. 1A through C). (**B**) Randomly chosen evolved *E. coli* PFM2 isolates (generation 650; *n* = 4) were grown for 24 h as monocultures (MDC with 10 mM glucose, 10 mM NH_4_Cl), with and without 50 μM adenine alongside ancestral PFM2 (Anc) and a *ΔpurH* mutant (*n* = 3). Each point represents a change in cell density for a single isolate between 0 and 24 h; replicate measurements were not made for any isolate. (**C and D**) RT-qPCR quantification of *purH* transcript levels in *E. coli* (*Ec*) PFM2 (**C**; relative to *hcaT*) and *R. palustris* (*Rp*) NifA* (CGA676) (**D**; relative to *fixJ*) in monoculture (mono) or coculture (co); 100 μM adenine (+ ade) was used to ensure that it was not used up before harvesting RNA. Bars, mean ± SD, *n* = 3–4. Statistically significant differences from the monoculture condition for each strain were determined using an unpaired two-tail *t*-test; ***, *P* < 0.001; ****, *P* < 0.0001. (**E**) Randomly chosen evolved-*R. palustris* isolates (generation 650) were grown as monocultures (MDC with 20 mM acetate and 10 mM NH_4_Cl), with and without 50 μM adenine alongside the ancestral strain (Anc; CGA676) (*n* = 4). Monoculture isolates were grown for 6 days, and coculture isolates were grown for 7 days due to inclement weather, each with a corresponding ancestral control. Each point represents a change in cell density for a single isolate between 0 and 6 or 7 days; replicate measurements were not made for any isolate. (**B and E**) Each line connects measurements for the same isolate grown with and without adenine to facilitate comparisons.

We designed our cocultures to be based on the reciprocal exchange of fermentation products and NH_4_^+^; characterization of these interactions is available elsewhere (20–24). We later discovered that *R. palustris* also externalizes the purine, adenine, that can be consumed by *E. coli* (25, 26). Given that *de novo* purine synthesis is energetically costly, we expected to observe *E. coli* purine synthesis LOF mutations in cocultures; adenine was amply available from *R. palustris* in long-term cocultures (31). However, no *E. coli* PFM2 purine biosynthesis gene (*pur*) mutations were observed in cocultures (Fig. 3A) nor were adenine auxotrophs observed among 40 coculture isolates screened (Fig. 3B). Unexpectedly, four *E. coli* monoculture isolates, two in each of replicates 18 and 20, appeared to be adenine auxotrophs, showing similar growth trends to the *ΔpurH* auxotroph control (Fig. 3B). It is unclear why adenine auxotrophs occurred in monocultures. Herein, we focus on why adenine auxotrophs were not enriched in coculture.

The BQH predicts that cross-feeding will lead to LOF mutations only when the benefit of cross-feeding outweighs the cost of maintaining the gene (2). To directly test whether adenine auxotrophs would have an advantage in coculture, we compared *E. coli* PFM2 growth versus that of a *ΔpurH* mutant, with and without adenine. The *E. coli ΔpurH* mutant required adenine for growth, but despite the availability of adenine, the *ΔpurH* mutant did not grow faster than the parent (Fig. S1A and B). We also directly compared the fitness of *E. coli* PFM2 versus a *ΔpurH* mutant by coinoculating them in coculture with *R. palustris* NifA* (CGA676). We used a range of initial frequencies to simultaneously test for possible coexistence by mutual invasion, where an equilibrium frequency can be extrapolated from the x-intercept by linear regression analysis (10, 32–34). Although there was poor linear correlation in the assay, the *E. coli ΔpurH* mutant did not increase in frequency relative to the parent (Fig. S1C). Thus, our results suggest that a *ΔpurH* mutant does not have a competitive advantage over *E. coli* PFM2 in the presence of adenine, thus verifying that there was no benefit for PFM2 adenine auxotrophs in coculture.

One reason why *E. coli* PFM2 adenine auxotrophs would not have an advantage in coculture is if adenine availability repressed gene expression, thereby decreasing gene cost for the ancestor. This would be analogous to the situation described above for *P. aeruginosa* siderophore genes (11–13). In support of this hypothesis, we previously saw that *E. coli* MG1655 had lower *pur* transcript levels in coculture (22). We verified that the same is true for *E. coli* PFM2 by RT-qPCR quantification of *purH* transcript; coculture PFM2 *purH* levels were 20% of those in monoculture (Fig. 3C). Adding adenine to monocultures resulted in a *purH* transcript level that was 12% of that observed without adenine, suggesting that the low expression in coculture was due to adenine availability (Fig. 3C).

*R. palustris* also had less *purH* transcript in coculture, 50% of that in monoculture (Fig. 3D). It is unclear how *E. coli* would influence *R. palustris purH* transcript levels. Regardless, it is curious that adenine availability combined with *purH* expression in monoculture did not lead to *R. palustris pur* LOF mutations (Table S1) or any adenine auxotrophs (Fig. 3E). We speculate that *R. palustris* lacks adenine uptake mechanisms. This would also help explain extracellular adenine accumulation (25, 31); poor uptake can be an important contributor to extracellular metabolite accumulation (35–37).

### Strain-specific adenine toxicity likely influences *E. coli* evolution

**Fig 4:**
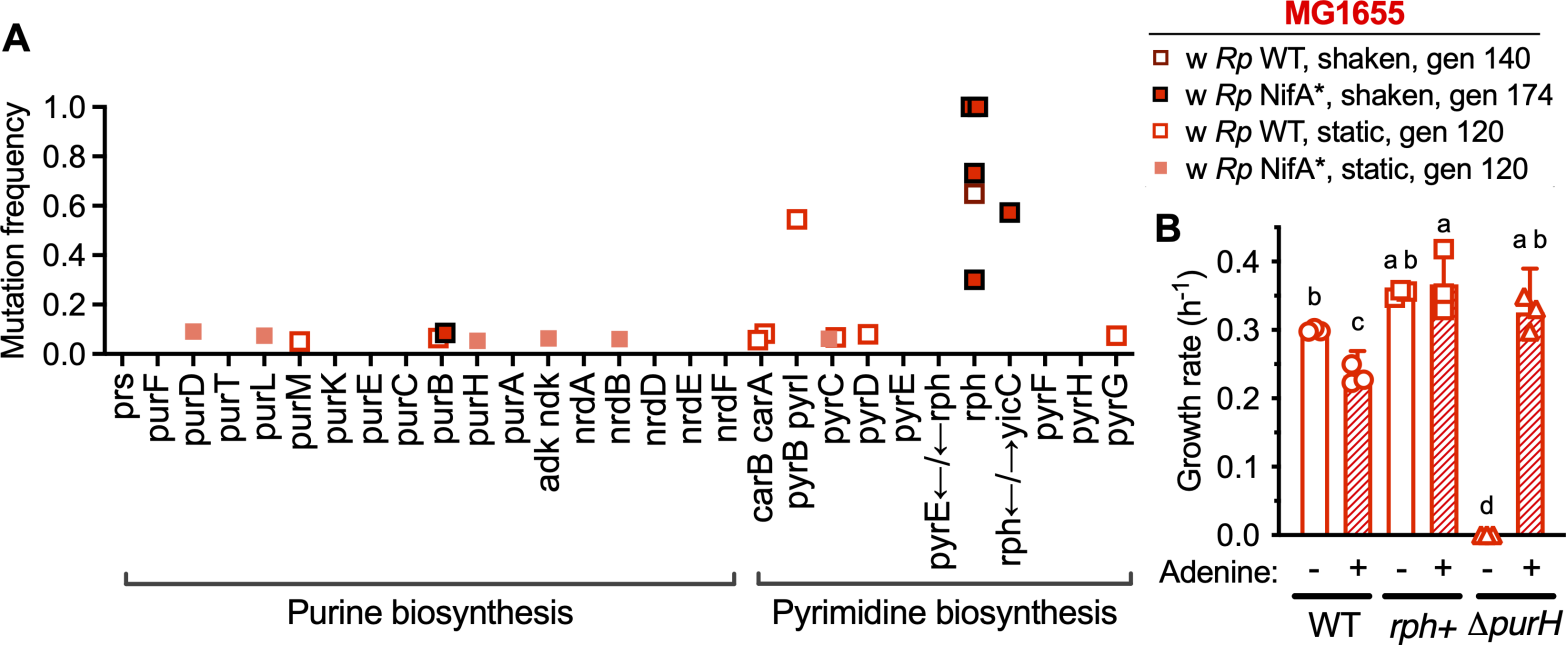
Adenine toxicity likely provides selective pressure for *E. coli* MG1655 *pur* mutations. (**A**) Each point represents an *E. coli* MG1655 mutation frequency in one replicate coculture (Fig. 1D and E). (**B**) *E. coli* MG1655 strains were grown as monocultures (MDC with 10 mM glucose, 10 mM NH_4_Cl), without (–) or with (+) 35 μM adenine. Letters indicate statistical differences (*P* < 0.05) as determined using a one-way ANOVA (*n* = 3).

Although cocultures did not enrich for *E. coli* PFM2 adenine auxotrophs, a low frequency of *pur* mutations was observed in previous cocultures featuring *E. coli* MG1655 (21) (Fig. 4A). We hypothesize that these mutations were enriched due to selective pressure from adenine toxicity. *E. coli* MG1655 is inhibited by adenine due to an *rph* mutation that disrupts *pyrE* regulation (38), leading to suboptimal precursor distribution between purine and pyrimidine synthesis in the presence of adenine (39). *E. coli* PFM2 does not experience this toxicity because the *rph* mutation is repaired (28, 29). In addition to the low frequency of *E. coli* MG1655 *pur* mutations in evolved cocultures, a high frequency of *rph* mutations was also observed (Fig. 4A). In contrast, *E. coli* PFM2 *rph* mutations were rare in monoculture and coculture (Fig. 3A; Table S2 through S4). We hypothesized that *E. coli* MG1655 *rph* and *pur* mutations alleviated adenine toxicity. Indeed, repairing the ancestral *rph* mutation or deleting *purH*, rendering *E. coli* MG1655 an adenine auxotroph, alleviated adenine toxicity (Fig. 4B). Thus, our results caution that toxic aspects of canonical biosynthetic precursors can enrich for mutations that might resemble those expected from the BQH.

### *R. palustris* siderophore gene mutations are correlated with expression level

**Fig 5:**
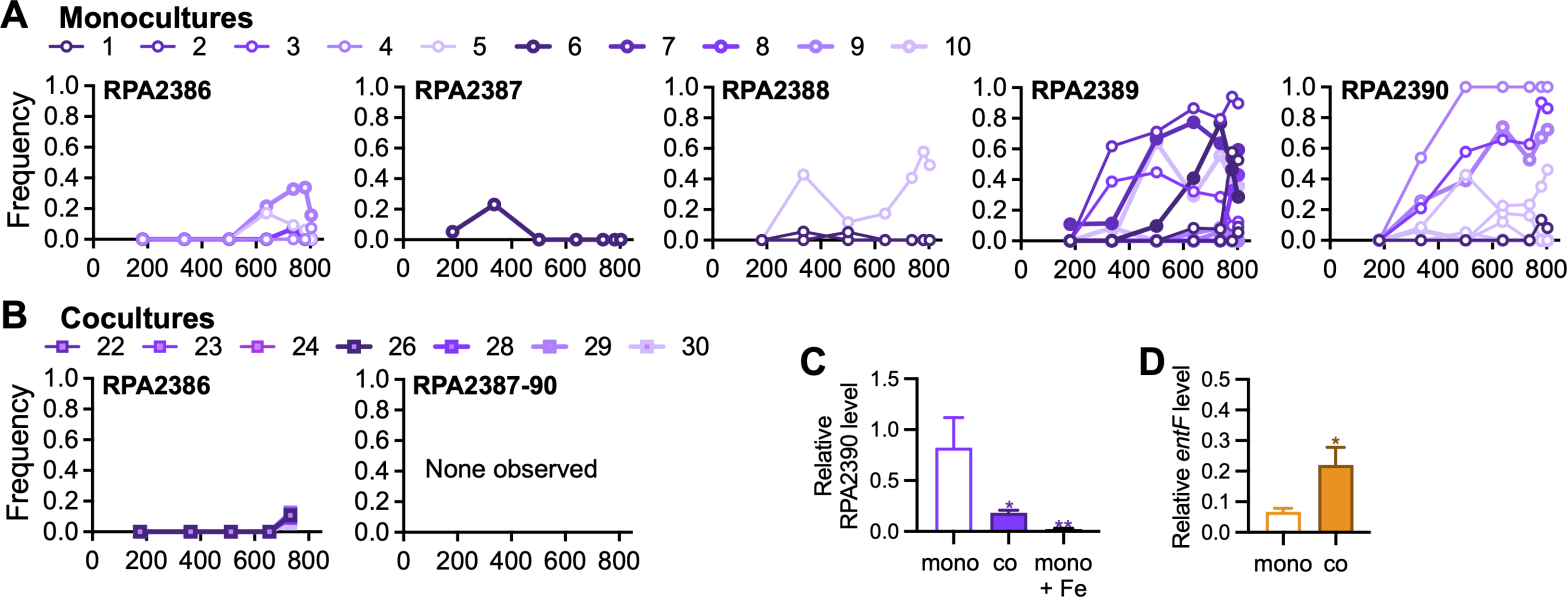
Siderophore gene loss is prevalent in *R. palustris* monocultures where gene expression is high. (**A and B**) *R. palustris* siderophore gene (RPA2386-90) mutation frequencies in monoculture (**A**) and coculture replicates (**B**). No mutations were observed in replicates 21, 25, and 27. Multiple lines with the same symbols in a given graph indicate different mutations in the same gene. (**C and D**) RT-qPCR quantification for a siderophore synthesis gene in *R. palustris* (**C**; RPA2390 relative to *fixJ*) and in *E. coli* (**D**; *entF* relative to *hcaT*). Values are the mean ± SD, *n* = 4. Statistically significant differences from the monoculture (mono) condition for each strain were determined using an unpaired two-tail *t*-test; *, *P* < 0.05; **, *P* < 0.01; co, coculture; Fe, ammonium ferric citrate.

A frequently mutated gene cluster in *R. palustris* monocultures was RPA2382-90 for siderophore synthesis (Fig. 2C, 5A and B). We speculate that these mutations gave rise to a subpopulation of “cheaters” of varying frequencies (Fig. 5A) that scavenged siderophores from the siderophore-producing subpopulation. However, we questioned why *R. palustris* siderophore mutants were not observed in cocultures.

One possibility for why siderophore gene mutations were not enriched in coculture could be if *E. coli* PFM2 facilitated *R. palustris* iron acquisition, thus decreasing the need for *R. palustris* to synthesize siderophores. This situation would be similar to *P. aeruginosa* siderophore mutants (11–13) and the case with adenine above, where low gene expression would lower gene cost and thereby lower the selection for LOF mutations. In support of this notion, we previously saw that *R. palustris* siderophore gene transcripts were low in coculture with *E. coli* MG1655, relative to monocultures (22). We verified that this trend was also true for *R. palustris* in coculture with *E. coli* PFM2; RT-qPCR analysis showed that the RPA2390 transcript level in coculture was 20% of that in monoculture (Fig. 5C). Adding soluble iron to monocultures decreased RPA2390 expression to 3% of that observed without added iron, suggesting that the low expression in coculture was in response to higher iron availability (Fig. 5C). Thus, iron must be scarce in *R. palustris* monocultures, inducing siderophore production, whereas in coculture, *E. coli* somehow increases iron availability, repressing *R. palustris* siderophore synthesis.

The mechanism of increased iron availability for *R. palustris* in coculture requires further investigation. However, we suspect that *R. palustris* can use *E. coli* siderophores. Others have speculated that *R. palustris* uses foreign siderophores because its genome encodes multiple siderophore transporters but only one synthesis cluster (40, 41). In support of this idea, the *E. coli* PFM2 siderophore *entF* transcript level was 3.3-fold higher in coculture than in monoculture (Fig. 5D), perhaps in response to iron loss to *R. palustris.*

### *E. coli* auxotrophs were observed in evolved monocultures

Perhaps similar to *R. palustris* siderophore mutant enrichment in monocultures, several *E. coli* PFM2 evolved isolates with unknown dependencies were observed in monoculture but not in coculture; 10 of the 40 evolved monoculture PFM2 isolates that we screened could not grow in a minimal medium (Fig. 3B). Four isolates were rescued by adenine, but the rest were not (Fig. 3B). Enrichment of auxotrophs in monoculture could suggest the emergence of cross-feeding between *E. coli* subpopulations that were not available in coculture, perhaps due to competition from an *R. palustris* population that is an order of magnitude larger (20, 21). Although we did not investigate these possible *E. coli* monoculture cross-feeding relationships, we speculate that a source of the auxotrophies could be mutations in *rpoC*, encoding the RNA polymerase β'-subunit. RpoC mutations are known to result in polyauxotrophs (42).

Curiously, we did not see a similar emergence of *R. palustris* auxotrophs (Fig. 3E). *E. coli* and other diverse bacteria are known to support, or quickly evolve to support, auxotrophic strains via cross-feeding (43–47). It is possible that *R. palustris* cannot support diverse auxotrophs beyond those that require adenine (25).

### *E. coli* PFM2 adenine transporter mutants are enriched in coculture

Here, we transition to possible gain-of-function mutations between conditions, starting with the possibility that coculture adenine availability influenced the emergence of adenine transporter mutations. For example, insertion element mutations were enriched in the intergenic region upstream of *adeD* encoding adenine deaminase (Fig. 2D). Insertion elements upstream of *adeD* in the adenine permease gene *adeQ* were previously observed to activate *adeD* expression (48).

**Fig 6:**
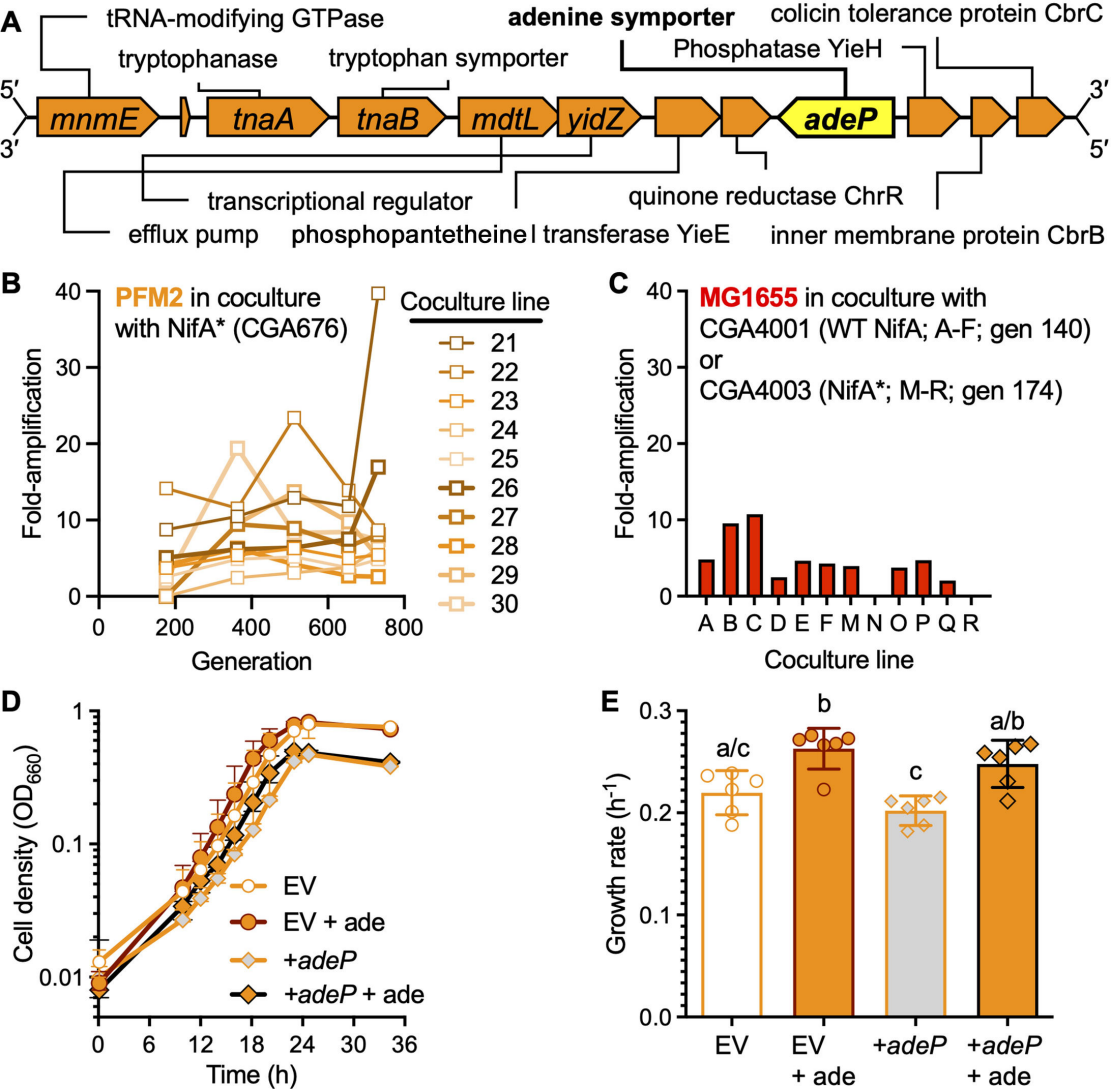
Amplification of *adeP* alone does not benefit *E. coli* PFM2 in the presence of adenine. (**A**) Consensus region of amplified *E. coli* genes observed in strains MG1655 and PFM2 in cocultures, but not in monocultures. The boundaries of the amplification differed between replicate cocultures, but each example always contained these genes. PFM2 is an *E. coli* MG1655 derivative with repaired *rpoS* and *rph* genes. (**B and C**) Fold-amplification (copy-number) of the genomic region shown in panel A for *E. coli* PFM2 (**B**) and *E. coli* MG1655 (**C**) replicate cocultures. Fold-amplification values were determined from sequencing coverage compared to the rest of the genome. (**D**) Effect of over-expressing *adeP* on *E. coli* PFM2 growth in monoculture (MDC with 10 mM glucose, 10 mM NH_4_Cl), with and without 35 μM adenine (ade). Over-expression was achieved by expressing *adeP* from the plasmid pCA24N with 30 μM IPTG. Data points are mean ± 95% C.I.; *n* = 3. EV, empty vector. (**E**) Growth rates from the same conditions as panel D. Bars, mean ± 95% C.I.; *n* = 6, combining two separate experiments. Different letters indicate statistical differences (*P* < 0.05) from a one-way ANOVA with Tukey’s multiple comparisons test.

Mutations were also enriched in the adenine permease gene, *adeP* (Fig. 2D). The *adeP* mutations were accompanied by amplification of a multi-gene region, ranging from 22 to 104 kB across replicate cocultures, with a 10 kb consensus region (Fig. 6A) that created up to 40 copies of *adeP* and neighboring genes (Fig. 6B). We revisited our previous long-term cocultures featuring *E. coli* MG1655 (21) and found similar mutations upstream of *adeD* and amplification of the same region (Fig. 6C). Evolved *E. coli* PFM2 monocultures did not show this amplification but had up to 8-fold amplification in another region, with no obvious connection to adenine (Fig. S2).

We hypothesized that these mutations could lead to increased fitness in the presence of adenine. We attempted to mimic a high *adeP* copy number via IPTG-induced *adeP* expression from a plasmid. However, over-expressing *adeP* in *E. coli* PFM2 did not improve growth in the presence of adenine over an empty vector control (Fig. 6D and E). It is possible that *adeP* amplification is only advantageous in the context of the surrounding amplified region or other enriched mutations.

To address an adenine-related fitness benefit more generally, we compared evolved *E. coli* PFM2 growth with and without adenine. Adenine benefited several coculture-evolved *E. coli* PFM2 isolates, suggesting that some mutations were adaptive to adenine availability (Fig. 3B). When isolates were treated as replicates, the change in cell density was significantly greater with adenine for coculture-evolved *E. coli* PFM2 isolates, compared to without adenine (unpaired two-tailed *t*-test, *P* = 0.0004). The trend was less pronounced for monoculture isolates, except for the four adenine auxotrophs (Fig. 3B), and there was no significant difference in the change in cell density between conditions with and without adenine when monoculture evolved isolates were treated as replicates (unpaired two-tailed *t*-test, *P* = 0.5480).

Although further characterization is necessary before conclusions can be made, our results suggest that adenine availability in coculture could have selected for gain-of-function mutants, which could involve transport. Elevated transporter expression is a potential cost against the emergence of LOF mutants, but one which can be offset if the savings from a LOF mutation is greater (49). Our results suggest such savings could also result from lower expression of purine synthesis genes, perhaps allowing for selection for mutants with enhanced transport. The potential expression costs of *adeP*, and the many other genes in the amplified region, are interesting to consider. If any of those genes are highly expressed, then the benefit must be large to compensate for the associated burden of expression.

### *R. palustris* monocultures evolved to utilize H_2_

**Fig 7:**
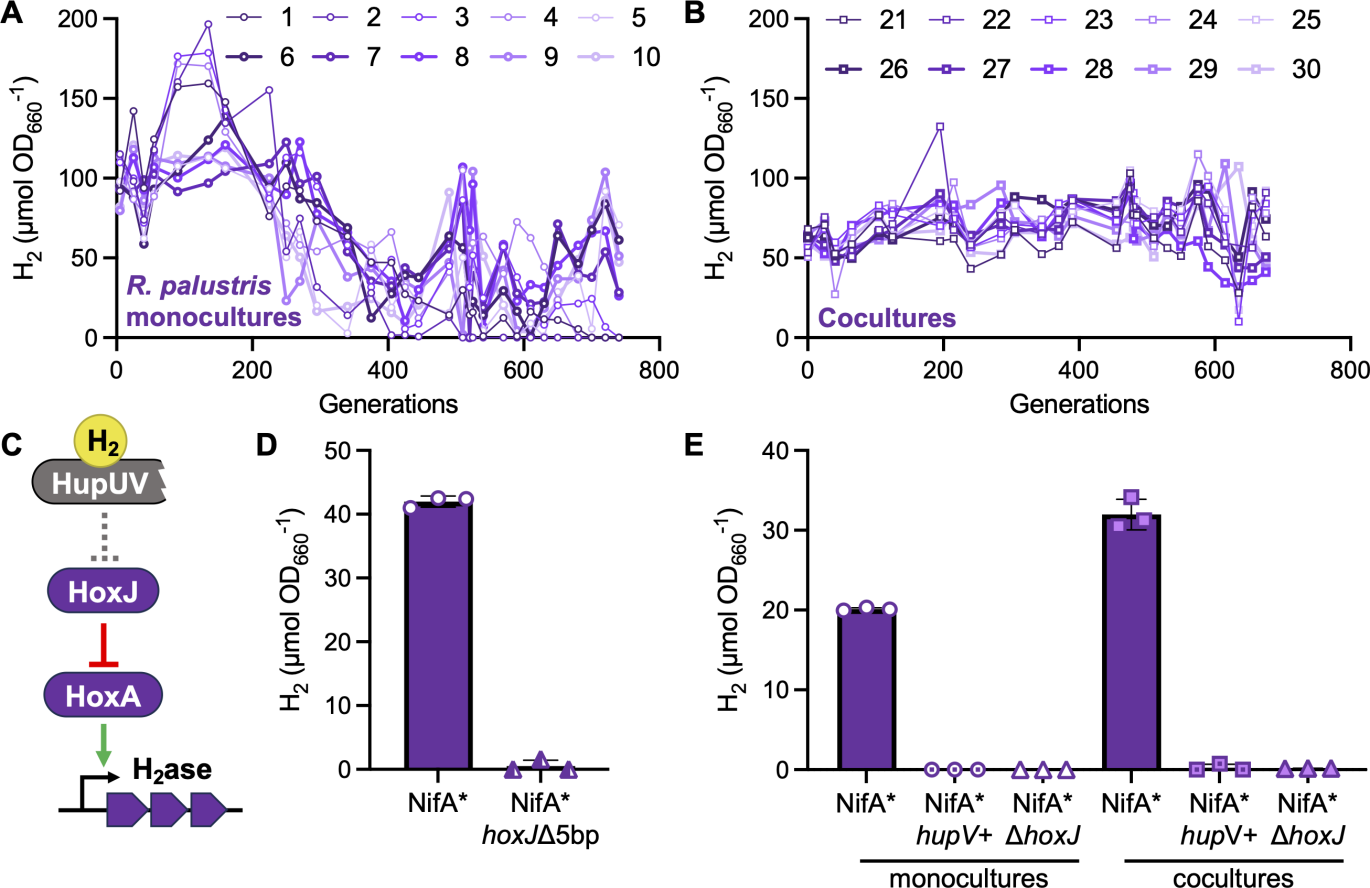
*R. palustris* monocultures evolve H_2_ utilization. H_2_ was quantified in 7-day-old cultures during serial transfers of *R. palustris* monoculture replicates 1–10 (**A**), and coculture replicates 21–30 (**B**). (**C**) *R. palustris* NifA* (CGA676) contains a *hupV* mutation that prevents the H_2_ sensor HupUV from repressing HoxJ. HoxJ then constitutively represses the transcriptional activator of hydrogenase (H_2_ase) genes, HoxA. (**D**) Stationary phase H_2_ in the NifA* ancestor without and with a 5-bp *hoxJ* deletion commonly observed in evolved monocultures. Cultures were grown in the same MDC medium used for monoculture serial transfers plus 1 µM NiSO_4_. (**E**) Stationary phase H_2_ levels in the NifA* ancestor without or with *hupV* repaired (*hupV*+) or *hoxJ* deleted. Monocultures were grown in MDC with 20 mM acetate and 10 mM NH_4_Cl. Cocultures were grown in the same media used for coculture serial transfers. (**D and E**) Bars, mean ± SD; *n* = 3.

We also explored an *R. palustris* gain-of-function mutation in monoculture. Both species produce H_2_, *E. coli* via hydrogenase during fermentation, and *R. palustris* as a coproduct of nitrogenase activity (50). Curiously, H_2_ levels decreased through serial transfers in *R. palustris* monocultures but not in cocultures (Fig. 7A and B). A commonly mutated gene in *R. palustris* monocultures was *hoxJ,* which encodes a sensor kinase that governs H_2_-oxidizing hydrogenase by repressing the transcriptional activator HoxA (Fig. 7C and 2C). Normally, HoxJ repression of HoxA is relieved when HupUV senses H_2_. However, the *R. palustris* ancestor had a *hupV* mutation, causing HoxJ to constitutively repress HoxA (51). Deleting HoxJ leads to hydrogenase activity (51).

Based on the above, we hypothesized that our *hoxJ* mutants also permitted H_2_ oxidation. To test this hypothesis, we introduced a 5-bp *hoxJ* deletion, observed in seven replicate monocultures, into the NifA* ancestor. Indeed, the mutant did not accumulate H_2_ (Fig. 7D). Thus, we reason that *hoxJ* mutations that were enriched in monoculture allowed *R. palustris* to use H_2_ as an electron source.

We questioned why *hoxJ* mutants were only enriched in monoculture. Previously, transposon disruption of *hoxJ* in monoculture led to a fitness benefit in stationary phase (52). The benefit likely stemmed from H_2_-mediated CO_2_ fixation after organic substrates were exhausted; strains with WT *hoxJ* could not grow after this point (52). In support of this observation, we saw a small, but non-significant increase in the growth rate when *hoxJ* deleted in *R. palustris* NifA* (Δ*hoxJ)*; cocultures with a NifA* Δ*hoxJ* mutant had a lower, but non-significant growth rate than those with the NifA* parent (Fig. S3).

To determine if there is something about coculture conditions that generally prevents H_2_ oxidation, we grew strains with a repaired *hupV* mutation (*hupV*+) or with Δ*hoxJ* mutation in monoculture versus coculture. In both conditions, no H_2_ accumulated when mutations enabled H_2_ oxidation, indicating that *R. palustris* hydrogenase can be activated in coculture (Fig. 4E). Therefore, something must have prevented enrichment of metabolically active *hoxJ* mutants in coculture.

Here, we discuss possibilities that could explain the lack of *hoxJ* mutant enrichment in coculture. First, consumable organic acids, which remained available at the time of transfer in cocultures, could have allowed the general *R. palustris* population to remain competitive against *hoxJ* mutants, preventing their enrichment. A second possibility is that *E. coli* outcompeted *R. palustris* for nickel, which is required for hydrogenase activity. Since our media used for serial transfers lacked a nickel supplement, nickel must have come as a contaminant in other media components, and its availability was likely low. Although the absence of H_2_ in cocultures with Δ*hoxJ* or *hupV*+ mutants (Fig. 7E) indicates that *R. palustris* can compete against *E. coli* for nickel, this might only be true when the H_2_-oxidizing *R. palustris* population is large. An emergent, low-frequency, H_2_-oxidizing *R. palustris* subpopulation might instead be outcompeted by *E. coli* for nickel. Although we assume that nickel was below the toxic range (53), it is also possible that *E. coli* nickel utilization for hydrogenase cross-protected ancestral *R. palustris* from nickel toxicity, negating any advantage of a nickel-utilizing *R. palustris hoxJ* mutant; in monocultures, *hoxJ* mutants could have played this role by sequestering nickel for hydrogenase.

### Concluding remarks

Long-term serial transfers of *R. palustris* and *E. coli* monocultures and cocultures differentially enriched for mutations. More differentially enriched mutations were observed in *R. palustris* monocultures compared to cocultures, and vice-versa for *E. coli*. Against expectations for cross-feeding to promote LOF mutations related to known or hypothesized adenine and iron availability via cross-feeding, *R. palustris* siderophore mutants (Fig. 5A) and *E. coli* adenine and unknown auxotrophs were only observed in evolved monocultures (Fig. 3B). No evolved dependencies that would prevent growth in a defined medium were observed in evolved cocultures (Fig. 3B and E). We reason that adenine and siderophore LOF mutants were not observed in coculture because cross-feeding generated metabolite levels that were sufficient to repress gene expression, thereby lowering gene cost; when gene cost is minimized, selective pressure for LOF mutations is also minimized, thus promoting gene retention. Our results also suggest that LOF mutations, resembling those expected from the BQH, can emerge from selective pressure from toxic side-effects of metabolites like adenine (Fig. 4). This situation might occur more frequently than expected; a large-scale survey of soil bacteria for auxotrophs omitted nucleotides from the defined medium after finding that nucleotides inhibited growth for many isolates (43). Separately, *E. coli* PFM2 acquired mutations that were suggestive of gain-of-function for adenine transport in coculture (Fig. 6), whereas *R. palustris* gain-of-function mutations enabling H_2_ utilization were only observed in evolved *R. palustris* monocultures (Fig. 7).

Our results, and those by others, suggest that the extent to which cross-feeding influences evolutionary landscapes depends on the organisms and conditions involved (54). The effects of cross-feeding on total mutation accumulation and on aspects of gene loss, retention, or gain-of-function mutations can be more nuanced than is often assumed. Adaptive evolution of synthetic communities can help expose and understand such nuances (49, 54, 55) and potentially exploit them to manipulate microbial communities to benefit society (56–58).

## MATERIALS AND METHODS

### Bacterial strains

*R. palustris* CGA676 is derived from CGA0092 (59) and carries a nifA* mutation that causes NH_4_^+^ excretion under N_2_-fixing conditions (20, 27). *R. palustris* mutants were made by homologous recombination using constructs in the suicide vector pJQ200SK (60). All primers are in Table S6. pJQhoxJΔ5bp was made by amplifying 1.4 kb from an evolved isolate from replicate A2 using primers JLM32 and JLM33, cutting with BamHI and XhoI, and then ligating into pJQ200SK, cut with the same enzymes. The ligation reaction was used to transform NEB10β for plasmid verification by PCR and Sanger sequencing. pJQhoxJΔ5bp was then used to transform *E. coli* S17-1 by electroporation. In each case, transformed *E. coli* was recovered on LB agar (Miller) with gentamycin (30 μg/mL^−1^). *E. coli* S17-1 with pJQ-ΔhoxJ and pJQ-hupV were gifts from C. S. Harwood (61).

R. palustris nifA* hoxJΔ5bp (CGA4065), nifA* Δ*hoxJ* (CGA4050), and nifA* *hupV*+ (CGA4062) were then made by introducing pJQhoxJΔ5bp, pJQ-ΔhoxJ*,* or pJQ-hupV, respectively, into CGA676 by conjugation with an *E. coli* S17-1 donor as described (61). Single recombinants were recovered on photosynthetic medium (PM) agar (62, 63), with 10 mM succinate and 100 μg/mL^−1^ gentamycin. Double recombinants were recovered by counterselection on PM succinate plates with 10% sucrose, screened for gentamycin sensitivity, and then verified by PCR amplification and Sanger sequencing.

*E. coli* PFM2 was a gift from P. Foster and is an *E. coli* MG1655 derivative with repaired *rpoS* and *rph* genes (28, 29). The Δ*purH* mutant was made via lambda Red recombination (64) using constructs amplified from the Δ*purH*::km^R^ KEIO mutant JW3970 (65) using primers YCC29 and YCC30. FLP-mediated excision was used to remove the km resistance cassette (64). All mutants were verified by PCR amplification and Sanger sequencing.

### Growth conditions

Colonies were recovered from 25% glycerol −80°C frozen stocks by streaking to LB agar (*E. coli*) or PM agar with 10 mM succinate. Single colonies were then used to inoculate starter cultures. Anoxic media in test tubes were prepared by bubbling N_2_ through 10 mL of media in 27-mL anaerobic test tubes, then sealing with rubber stoppers and aluminum crimps prior to autoclaving. Monocultures and cocultures were grown horizontally at 30°C with light from a 45 W halogen bulb (430 lumens) and shaking at 150 rpm in minimal M9-derived coculture medium (MDC) (20), with 10 mM glucose, 10 mM NH_4_Cl, and cation solution (100× stock: 100 mM MgSO_4_ and 10 mM CaCl_2_) for *E. coli* monocultures; 20 mM sodium acetate and 10 mM NH_4_Cl for *R. palustris* monocultures; or 25 mM glucose and cation solution for cocultures. Plasmid-carrying strains were also supplemented with 100 μg/mL gentamycin or 25 μg/mL chloramphenicol as appropriate. Starter cultures were inoculated with single colonies. *R. palustris* starter cultures were grown in MDC with 20 mM acetate and 10 mM NH_4_Cl. *E. coli* starter cultures were grown aerobically in LB, with 30 μg/mL kanamycin (km) when appropriate. *E. coli* starter cultures were washed twice in 1 mL MDC prior to inoculating test cultures or bioassays. Cocultures were inoculated with 0.1 mL each of *R. palustris* and *E. coli* to an initial optical density (OD_660_) of ~0.003 each.

Cultures in 96-well plates were treated similarly except that oxic solutions were used to prepare 0.2-mL volumes in each well. Anoxic conditions were achieved by sealing plates inside a BD GasPak EZ large incubation container with anaerobic sachets.

### Experimental evolution

Founder monocultures of *E. coli* PFM2 and *R. palustris* NifA* CGA676 were each grown from a single colony in anoxic MDC with either 10 mM glucose, cation solution, and 3 mM NH_4_Cl for PFM2 or 20 mM sodium acetate for CGA676. A single-founder monoculture was then used to inoculate 10 monocultures and 10 cocultures. All cultures were grown horizontally without shaking at 30°C with light in MDC. *E. coli* PFM2 monocultures were supplemented with 10 mM glucose, cation solution, 25 mM NaCl, and 2.3 mM NH_4_Cl. These cultures were started later, after finding that 25 mM glucose led to periodic extinction due to prolonged exposure to acidic fermentation products and concerns that comparative analyses would be hampered by the strong selection for acid-resistant mutants in monocultures. As a result, *E. coli* monocultures were transferred fewer times than for other conditions. No data from monocultures with 25 mM glucose were presented herein. CGA676 monocultures were supplemented with 25 mM glucose, cation solution, 8 mM disodium succinate, 7.3 mM sodium acetate, 0.25 mM sodium formate, 1.4 mM sodium lactate, and 6.3 mM ethanol; glucose was provided for consistency with other conditions but *R. palustris* cannot consume glucose. Cocultures were supplemented with 25 mM glucose, cation solution, and 25 mM NaCl; acidification was not a problem despite using 25 mM glucose because *R. palustris* consumes enough organic acids to dampen the pH drop (20). Every 7 days, cultures were vortexed, and 0.25 mL was transferred to fresh medium. About every five transfers, stocks were diluted 1:1 with 50% glycerol and stored at −80°C. Separately, cell pellets from 1 mL samples were frozen for gDNA extraction. Experimental evolution conditions for cocultures with *E. coli* MG1655 (Fig. 1D and E) are described elsewhere (21).

### Analytical procedures

Cell densities were measured via turbidity (OD_660_) using a Genesys 20 visible spectrophotometer (Thermo Fisher). H_2_ was measured using a Shimadzu GC-2014 gas chromatograph with a thermal conductivity detector as described by sampling 0.1 mL of headspace using a gas-tight syringe (66). Statistical analyses were performed using GraphPad Prism (v10).

### Competition assays

Competition assays were conducted in an invasion-from-rare format to consider coexistence by the mutual invasion criterion, where each population can increase when rare (32, 34). Cocultures were started from various initial frequencies (targeting 0.01–0.99) of each *E. coli* strain (WT vs Δ*purH* or WT vs. Δ*purH*::km^R^) for a total initial cell density of ~10^6^ colony-forming units (CFU)/mL. *R. palustris* CGA676 was inoculated to an initial density of ~10^6^ CFU/mL. Frequencies were determined upon inoculation and after 5 days. WT and Δ*purH* mutants were distinguished by counting CFUs on M9 agar with cations and 25 mM glucose and on LB agar and then determining Δ*purH* populations from the difference. Change in frequency = [*E. coli* Δ*purH* / (*E. coli* WT + *E. coli* Δ*purH*)]_final_ – [*E. coli* Δ*purH*/(*E. coli* WT + *E. coli* Δ*purH*)]_initial_ (33).

### Genome sequencing and mutation analysis

gDNA was purified using a Qiagen DNeasy Blood and Tissue kit following the manufacturer’s instructions. Lysis was facilitated after resuspension by adding proteinase K (50 μg/mL final) and incubating at 56°C for 10 min. RNase A (4 μL, Promega) was then added, and the lysate was incubated for 2 min before proceeding.

DNA fragment libraries were made using a NextFlex Bioo Rapid DNA kit, and libraries were sequenced using Illumina NextSeq 500 150 × 150 paired-end runs by the IU Center for Genomics and Bioinformatics. Paired-end reads were pre-processed for quality with cutadapt 3.4 (67) with the following options: -a AGATCGGAAGAGC -A AGATCGGAAGAGC; -q 15,10; -u 6. Mutations were called using breseq v. 0.32.0 on polymorphism mode (68). *E. coli* monoculture population sequences were mapped to the MG1655 genome (accession NC_000913); *R. palustris* monoculture population sequences were mapped to a concatenated reference genome consisting of the CGA009 chromosome (accession BX571963), and its plasmid pRPA (accession BX571964). Co-culture sequences were mapped to a concatenation of the *E. coli* and *R. palustris* reference genomes. Polymorphisms that co-occurred in both the monoculture and co-culture data sets were filtered and maintained as a subset to enrich for the most informative variants representing treatment differences (Tables S1 through S4). This filtering step also removed sequence differences between the reference sequences and those of the experimental strains used. Variants were prioritized as mutations of interest if they were detectable at the final two sequencing time points and co-occurred across multiple populations in the same locus. All mutations are in Tables S1 through S4. Locus tag conversions are in Table S5.

### Reverse-transcription quantitative real-time PCR (RT-qPCR)

Cultures received 100 μM adenine or ammonium ferric [iron(III)] citrate as indicated. Cultures were harvested in exponential phase at 0.6–0.8 OD_660_ except for *E. coli* monocultures (+/– adenine experiment), which were harvested at 0.3–0.4 OD_660_ to avoid adenine depletion. Cultures were chilled on ice and pelleted by centrifugation. Lysis, RNA purification, and cDNA generation were performed exactly as described (25). Standard curves were generated using gDNA. Transcripts were quantified as described (25) using the appropriate primers (Table S6) with a Mastercycler ep *realplex* real-time PCR system (Eppendorf). Data were analyzed by *realplex* software using Noiseband. Specificities were validated by melting curves and by the presence of a single band on an agarose gel.

## Data Availability

Genome sequence reads are in the NCBI Sequence Read Archive under BioProject accession number PRJNA1079325 (https://www.ncbi.nlm.nih.gov/bioproject). All other data for this study are in this article and its supplemental material.

## SUPPLEMENTAL MATERIAL

Supplemental material aem.00363-26-s0001.pdf Supplemental text, Table S6, and Fig. S1 to S3.

Supplemental tables aem.00363-26-s0002.xlsx Tables S1 to S5.

ASM does not own the copyrights to Supplemental Material that may be linked to, or accessed through, an article. The authors have granted ASM a non-exclusive, world-wide license to publish the Supplemental Material files. Please contact the corresponding author directly for reuse.
