# Supplementary material for: Reciprocal cross-feeding between bacteria can limit the emergence of metabolic dependencies
Source: Appl Environ Microbiol. 2026 May 12;92(6):e00363-26. doi: 10.1128/aem.00363-26 (PMC13274399; doi:10.1128/aem.00363-26)
Supplement: Supplemental material — Supplemental text, Table S6, and Fig. S1 to S3. [file aem.00363-26-s0001.pdf]

SUPPLEMENTARY MATERIALS FOR

Reciprocal cross-feeding between bacteria can limit the emergence of metabolic dependencies

Ying-Chih Chuang[1,2,†], Megan G. Behringer[3], Gillian Patton[3], Jordan T. Bird[4], Jeffrey L. Mazny[1], Jennifer R. Gliessman[1], Crystal E. Love[1], Ankur Dalia[1], and James B. McKinlay[1*]

[1]Department of Biology, Indiana University, Bloomington, IN, USA
[2]Biochemistry Program, Indiana University, Bloomington, IN, USA
[3]Department of Biological Sciences, Vanderbilt University, Nashville, TN, USA
[4]Battelle Memorial Institute, Columbus, OH, USA

*Corresponding author: 1001 E 3rd Street, Bloomington, IN 47405, USA;
Email: jmckinla@iu.edu

† Current address: Department of Biological Sciences, University of Southern California, Los Angeles, CA, USA

CONTENTS:

Supplemental text

Table S6. Primers

Fig S1. Adenine availability does not provide a fitness advantage to an engineered *E. coli* PFM2 purine auxotroph.

Fig S2. *E. coli* PFM2 gene amplification region in monocultures

Fig S3. An H_2 oxidizing Δ*hoxJ* mutant does not have a higher growth rate in monoculture or coculture.

References for Supplementary materials

Supplemental Text

Brief insights into other differentially enriched mutations. As in previous long-term cocultures pairing a different *R. palustris* NifA* strain with *E. coli* MG1655 (1), several *E. coli* PFM2 mutations in coculture suggested adaptation to low-nitrogen; in coculture, the *E. coli* growth rate is limited to ~20% of maximum due to the limiting NH_4^+ supply from *R. palustris* (1, 2). Mutations were commonly observed in *glnGL,* encoding the two-component system NtrBC that controls nitrogen scavenging. Previously, we demonstrated that NtrBC is critical for *E. coli* survival in the low-nitrogen coculture environment (1, 3). Similar mutations were also enriched in nitrogen-limited *E. coli* monocultures by another group (4). Other mutations in *glnK* and *amtB*, which respectively encode a nitrogen-responsive regulatory protein and an NH_4^+ transporter, likely also help with nitrogen acquisition in coculture (Fig 6); we also previously demonstrated the importance of *E. coli* AmtB in coculture (5).

Several *E. coli* PFM2 genes mutated in coculture might also impact biofilm formation and acid sensitivity. We speculate that these mutations could help *E. coli* associate with *R. palustris* and/or *E. coli* cross-feeding partners and potentially respond to fermentative acid stress. For example, *csgD/B* is involved in curlin-based biofilm formation (6), which is regulated in part by *cpxRP* genes that were also mutated (7). *ycgV* encodes an outer membrane protein that might be involved in biofilm formation and *fimH/A* are involved in fimbriae-mediated adhesion (6). Mutations in *maoP*/*hdfR*, might also affect biofilm formation. MaoP affected biofilm formation in addition to its role in chromosome positioning and segregation (8) and HdfR might have a regulatory link to the transition

between motile and sessile lifestyles (9). HdfR might also be linked to acid resistance by regulating amino acid decarboxylation genes encoded by *gltBD (10). E. coli* MG1655 *hdfR* mutations were also previously observed in evolved cocultures (1). *E. coli* PFM2 mutations were also observed in *cspC*/*yobF*, which encode for proteins that respond to acid and other stressors (11). Some of the genes associated with the amplified region in cocultures might also be linked to acid resistance such as *mnmE* (12) and *yidZ* (13).

Other differentially enriched *E. coli* PFM2 mutations include those in *aceE*, observed in evolved monocultures (Fig 6). *aceE* encodes a pyruvate dehydrogenase subunit, which is important for respiration but is generally not involved in *E. coli* fermentative metabolism (14). It is possible that expression of *aceE* is sub-optimally high under the high growth rates supported in monoculture conditions (3). Other mutations enriched in monoculture occurred in genes for post-translational regulation of isocitrate dehydrogenase (*aceK*), ribosome modification (*rluC*), a vitamin C transporter (*ulaA/B*), and a deoxyribose salvage enzyme (*deoB*).

One of the few *R. palustris* genes that was differentially mutated in at least 5 replicate cocultures was RPA3297, annotated to encode a urea or short-chain amino acid transporter. Each mutation caused a frame shift and thus they are all likely LOF mutations. This transporter was previously observed to have elevated protein levels in coculture compared to monoculture (3). We speculate that this elevated expression was disadvantageous, but the function of the gene product remains unknown.

Two replicate *R. palustris* cocultures also had an intergenic mutation between *hbdA* and *etfA*. These genes are likely involved in fatty acid degradation and might be commonly regulated by LiuR (15). The effect of this mutation is unknown. Genes that were mutated in monoculture include a methylmalonyl-CoA mutase encoded by RPA1837, a possible flagellin encoded by RPA2297 that is outside the flagellar gene cluster, and a hypothetical protein encoded by RPA4084 (6 of 10 replicates). Potential effects of these mutations are unknown.

***R. palustris* genes that were commonly mutated in monoculture and coculture.** Several differentially enriched mutations in *R. palustris* occurred in common genes between monocultures and cocultures, suggesting a general benefit. Among these was *ppsR2*, encoding an O_2-responsive regulator of genes for phototrophic growth. *ppsR2* mutations are known to provide an advantage under low-light conditions, which occurs at high cell densities (16). Other genes that were commonly mutated in monoculture and coculture were *cbbM*, encoding the Calvin cycle type II Rubisco and *cbbR*, encoding a Calvin cycle regulator. It is unclear why these mutations were enriched, but it is possible that they are associated with the role of CO_2 fixation in electron balance during photoheterotrophic growth on electron-rich fermentation products or electron distribution between CO_2 and N_2 fixation, potentially affecting NH_4^+ excretion (17, 18). We did not investigate *ppsR2* and Calvin cycle gene mutations herein, choosing instead to focus on genes and regions that were differentially mutated between monocultures and cocultures.

Table S6. Primers.

Primer	Sequence (5'-3')	Description
JLM32	tagtggatccgctcaccgatctcgtcgatc	Forward *ΔhoxJ*5bp (BamHI)
JLM33	actgCTCGAgagtagcggtcggacgttc	Reverse *ΔhoxJ*5bp (XhoI)
YCC29	gcgcaaacgttttcgttacaatgcg	5' of Δ*purH*::*Km* in JW3970
YCC30	tgcattacccggagcaac	3' of Δ*purH*::*Km* in JW3970
YCC80	taagggaaccgtgcatgtg	Forward qPCR primer for *Rp fixJ*
YCC81	ggattcgtacagcttgacctc	Reverse qPCR primer for *Rp fixJ*
YCC97	acgtcgtcgtggttcttg	Forward qPCR primer for *Rp purH*
YCC98	cgaagccaccgtcgataaa	Reverse qPCR primer for *Rp purH*
YCC99	gcaacacgttctgctgatg	Forward qPCR primer for *Rp RPA2390*
YCC100	cattggttctcggcctatct	Reverse qPCR primer for *Rp RPA2390*
YCC91	aaccgcatggcccttatt	Forward qPCR primer for *Ec entF*
YCC92	gtatccagcaagccaagaaatg	Reverse qPCR primer for *Ec entF*
YCC93	gtgttcgaaggctttgatgg	Forward qPCR primer for *Ec purH*
YCC94	gtgaagagcagcgactatga	Reverse qPCR primer for *Ec purH*
YCC95	gtcgcactttgccgtaatc	Forward qPCR primer for *Ec hcaT*
YCC96	gctgatgctggtgatgattg	Reverse qPCR primer for *Ec hcaT*

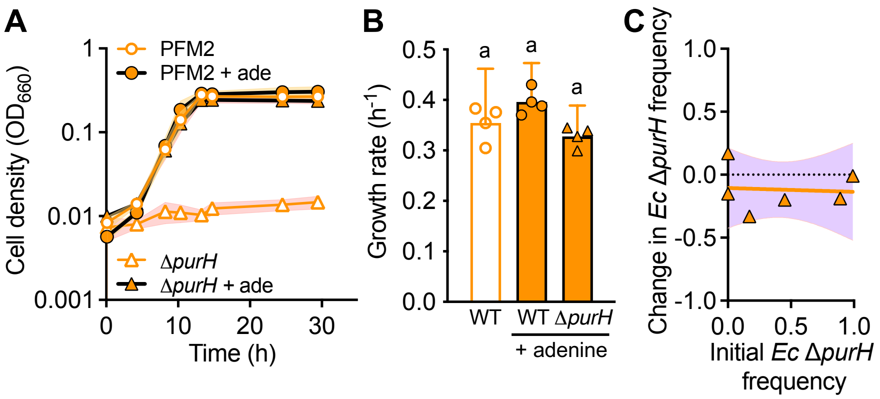

Fig S1. Adenine availability does not provide a fitness advantage to an engineered *E. coli* PFM2 purine auxotroph. (A) Growth of WT *E. coli* PFM2 and its *ΔpurH* mutant as monocultures (MDC with 10 mM glucose, 10 mM NH_4Cl, and cations), with and without 35 μM adenine (ade)**.** Points, mean ± SD as shading; n=3. **(B)** Monoculture growth rates for *E. coli* PFM2 (WT) vs a corresponding *ΔpurH* mutant with and without 35 μM adenine. 'a,' indicates statistically similar values ($p > 0.01$) as determined using a one-way ANOVA with Tukey's multiple comparisons test. Bars, mean ± SD; n=3. **(C)** Invasion-from-rare assay competing an *E. coli ΔpurH* mutant against its PFM2 parent in coculture with *R. palustris* NifA* (CGA676) under N_2-fixing conditions where *R. palustris* excretes NH_4^+ and adenine. The orange line is the best fit from a linear regression analysis with 95% CI shaded in purple. Change in frequency = (*E. coli* Δ*purH* / (*E. coli* WT + *E. coli* Δ*purH*))$_{final}$ – (*E. coli* Δ*purH* / (*E. coli* WT + *E. coli* Δ*purH*))$_{initial}$. **(B, C)** Each data point represents a measurement from a single biological replicate.

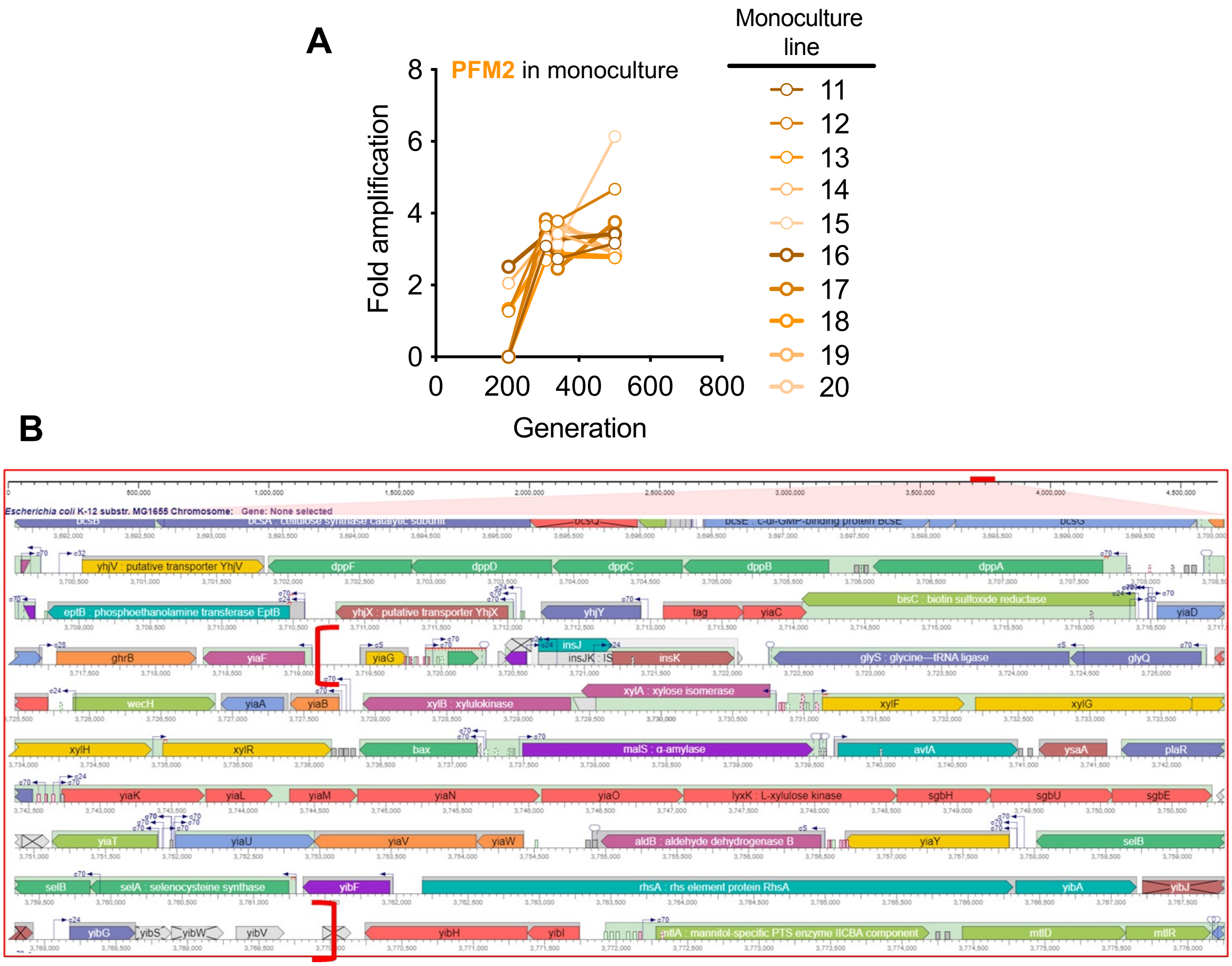

Fig S2. *E. coli* PFM2 gene amplification region in monocultures. (A) Level of amplification observed in PFM2 monocultures. **(B)** Amplified region of the chromosome based on mapping to *E. coli* MG1655. Image was generated using Metacyc.org.

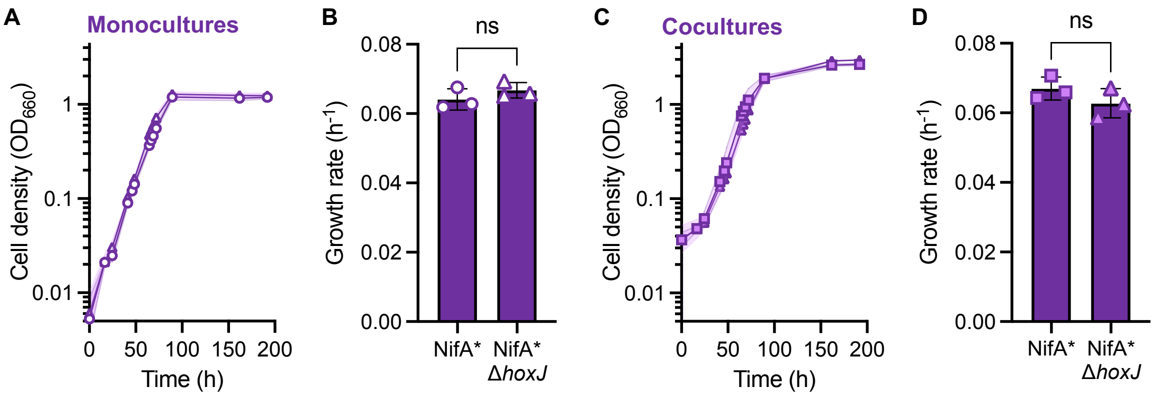

Fig S3. An H_2 oxidizing Δ*hoxJ* mutant does not have a higher growth rate in monoculture or coculture. Monoculture growth curves **(A)** and exponential growth rates **(B)** of *R. palustris* NifA* (CGA676) and its Δ*hoxJ* mutant. Growth curves **(C)** and exponential growth rates **(D)** for cocultures pairing *E. coli* PFM2 with either *R. palustris* NifA* (CGA676) or its Δ*hoxJ* mutant. Monocultures were grown in MDC with 20 mM acetate and 10 mM NH_4Cl. Cocultures were grown in same media used for coculture serial transfers. **(A, C)** Line is the mean ± SD as shading; n=3. **(B, D)** Bars, mean ± SD; n=3. ns, no significant difference determined using an unpaired two-tail t-test.

References for the supplementary materials

1. Fritts RK, Bird JT, Behringer MG, Lipzen A, Martin J, Lynch M, McKinlay JB. 2020. Enhanced nutrient uptake is sufficient to drive emergent cross-feeding between bacteria in a synthetic community. ISME J 14:2816–28.
2. LaSarre B, McCully AL, Lennon JT, McKinlay JB. 2017. Microbial mutualism dynamics governed by dose-dependent toxicity of cross-fed nutrients. ISME J 11:337-48.
3. McCully AL, Behringer MG, Gliessman JR, Pilipenko EV, Mazny JL, Lynch M, Drummond DA, McKinlay JB. 2018. An *Escherichia coli* nitrogen starvation response Is important for mutualistic coexistence with *Rhodopseudomonas palustris*. Appl and Environ Microbiol 84:e00404-18.
4. Warsi OM, Andersson DI, Dykhuizen DE. 2018. Different adaptive strategies in *E. coli* populations evolving under macronutrient limitation and metal ion limitation. BMC Evol Biol 18:72.
5. McCully AL, LaSarre B, McKinlay JB. 2017. Recipient-biased competition for an intracellularly generated cross-fed nutrient is required for coexistence of microbial mutualists. mBio 8:e01620-17.
6. Beloin C, Roux A, Ghigo JM. 2008. *Escherichia coli* biofilms. Curr Top Microbiol Immunol 322:249-89.
7. Prigent-Combaret C, Brombacher E, Vidal O, Ambert A, Lejeune P, Landini P, Dorel C. 2001. Complex regulatory network controls initial adhesion and biofilm formation in *Escherichia coli* via regulation of the *csgD* gene. J Bacteriol 183:7213-23.
8. Holden ER, Yasir M, Turner AK, Wain J, Charles IG, Webber MA. 2021. Massively parallel transposon mutagenesis identifies temporally essential genes for biofilm formation in *Escherichia coli*. Microb Genom 7:000673.
9. Barker CS, Pruss BM, Matsumura P. 2004. Increased motility of *Escherichia coli* by insertion sequence element integration into the regulatory region of the flhD operon. J Bacteriol 186:7529-37.
10. Krin E, Danchin A, Soutourina O. 2010. Decrypting the H-NS-dependent regulatory cascade of acid stress resistance in *Escherichia coli*. BMC Microbiol 10:273.
11. Hobbs EC, Astarita JL, Storz G. 2010. Small RNAs and small proteins involved in resistance to cell envelope stress and acid shock in *Escherichia coli*: analysis of a bar-coded mutant collection. J Bacteriol 192:59-67.
12. Sayed AK, Foster JW. 2009. A 750 bp sensory integration region directs global control of the *Escherichia coli* GadE acid resistance regulator. Mol Microbiol 71:1435-50.
13. Gao Y, Lim HG, Verkler H, Szubin R, Quach D, Rodionova I, Chen K, Yurkovich JT, Cho BK, Palsson BO. 2021. Unraveling the functions of uncharacterized transcription factors in *Escherichia coli* using ChIP-exo. Nucleic Acids Res 49:9696-9710.
14. Snoep JL, de Graef MR, Westphal AH, de Kok A, Teixeira de Mattos MJ, Neijssel OM. 1993. Differences in sensitivity to NADH of purified pyruvate dehydrogenase complexes of *Enterococcus faecalis*, *Lactococcus lactis*, *Azotobacter vinelandii* and *Escherichia coli*: implications for their activity in vivo. FEMS Microbiol Lett 114:279-83.

15. Kazakov AE, Rodionov DA, Alm E, Arkin AP, Dubchak I, Gelfand MS. 2009. Comparative genomics of regulation of fatty acid and branched-chain amino acid utilization in proteobacteria. J Bacteriol 191:52-64.
16. Fixen KR, Harwood CS. 2016. A polymorphism in the oxygen-responsive repressor PpsR2 confers a growth advantage to *Rhodopseudomonas palustris* under low light. Photosynth Res 129:199-204.
17. McKinlay JB, Harwood CS. 2010. Carbon dioxide fixation as a central redox cofactor recycling mechanism in bacteria. Proc Natl Acad Sci U S A 107:11669-75.
18. McKinlay JB, Harwood CS. 2011. Calvin cycle flux, pathway constraints, and substrate oxidation state together determine the H_2 biofuel yield in photoheterotrophic bacteria. mBio 2:e00323-10.
